# Extracts

**Published:** 1859-02

**Authors:** 


### EXTRACTS.

### A SUCCESSFUL CASE OF TRANSFUSION IN YELLOW FEVER.

To be the means of saving the life of a fellow-being evidently sinking into the grave—to do this by the application of principles of treatment essentially new in the particular disease subjected to treatment, and especially by the application of these principles to save the life of one who is as dear to the practitioner as his own life, is the very culmination of the ambition of the conscientious physician. Such has been the happy distinction attained by our friend, Dr. N. B. Benedict, of New Orleans, in a case published in the January number of the *New Orleans Medical News and Hospital Gazette.*

The epidemic of yellow fever which prevailed in New Orleans the past summer, was unusually fatal. Dr. B. had four cases of the disease in his own family, and it seemed as if one of them certainly had been marked by death as its victim; but a timely resort to transfusion called the patient back to life again, and, we hope, *established* a new principle in the treatment, not only of yellow fever, but of other diseases where death seems to be inevitable from exhausting discharges.

The case, in brief, is as follows: Miss J. B., who had resided for nine years in Mississippi, and for four years in New Orleans, had suffered from no serious illness for fourteen years. On the 11th of October last, she was exposed to a drenching rain, and at 9 o'clock P. M., of the following day, was seized with a violent chill. The symptoms of yellow fever could not have been more characteristic than in this case, and it proved to be unusually obstinate during the first stage. On the third day Dr. C. B. White was called in, and on the fifth day Dr. Wm. E. Kennedy was added to the consultation. Hemorrhage from the mouth began on the fifth day and gradually increased, until, on the night of the eighth day, the blood, diluted with the saliva, saturated two sheets, so that no part of them remained unsoiled. A hemorrhagic tendency was also manifested at this time by other mucous surfaces besides those of the mouth. From the close of the ninth day, the hemorrhage began to diminish, and entirely ceased on the night of the tenth; but the complexion was much blanched, and the mental condition was that of utter despair as to recovery. The rate of the pulse was steadily accelerated, the average for the seventh day being 98, for the eighth and ninth days 104, for the tenth day 115, and for the eleventh and twelfth days 120.

The tenth, eleventh and twelfth days were characterized by morbid nervous sensations, and mental vagaries. About noon of the thirteenth day, she was suddenly seized with vomiting, which continued throughout the day and the following night, nothing being retained by the stomach but a very little brandy; injections of beef-tea, brandy, and carbonate of ammonia were not retained; the sinking and prostration were excessive, and the bellows sound characteristic of anæmia was heard over the whole region of the aorta. At every movement of the body, distressing hiccough occurred, convulsing the whole frame, and lasting, on each occasion, about a minute. On the morning of the fourteenth day, a stimulating enema was followed by a discharge resembling coffee grounds, the mortal restlessness increased, the pulse became the merest flutter, and was much of the time inappreciable, and its number could not be ascertained.

On that day, at the morning consultation, Drs. Kennedy and White declared their conviction that the patient could not possibly survive for more than three or four hours. At this juncture Dr. B. said:

"It is an old saying that 'drowning men catch at straws.' For the past twenty-four hours I have been in torture with the thought of such a straw, and I cannot refrain from naming it: I mean transfusion. These fatal symptoms being occasioned by the loss of the last few ounces of blood, I cannot persuade myself that it is not a serious duty to inject into her veins a few ounces of fresh, healthy, living blood, as nearly as possible identical with that which she has lost."

The suggestion was promptly embraced, as the only thing that could possibly save life, and Dr. B. fortunately possessing an instrument for the purpose, it was immediately carried into execution with the aid of Drs. C. C. Beard, D. W. Brickell, and L. Greenleaf. Dr. B. describes the operation as follows:

"The apparatus was examined, its use described and illustrated, and every part of the process was fully explained and discussed. The task of preparing the vein was undertaken by Prof. C. C. Beard. An incision about two inches in length was made over the median vein in the left arm. A director was passed beneath the vessel, near the lower part of the incision, in order that it might be held under perfect control, and the loss of any blood from its distal extremity be prevented. An incision was then made into the vein, immediately beyond the director, to receive the beak of the syringe. It is due to Prof. Beard to say that nothing could exceed the skill and steadiness with which this operation was performed. The blood was obtained, by the assistance of Dr. Greenleaf, from the arm of a young gentleman who exhibits a remarkable example of perfect health, and who had experienced yellow fever during the epidemic of 1853.

"It was my purpose to use the apparatus myself; but as the moment approached, my near relationship to the patient made me distrust my firmness, and I requested Prof. Brickell to take my place. He consented to do so, but substituted for the syringe belonging to the apparatus one of simpler construction, into the beak of which he absorbed the blood as it flowed into the receiver of the transfuser. By inverting the syringe and pushing upward the piston, the last bubble of air was expelled. The beak was then introduced, by Dr. White, into the orifice of the exposed vein of the patient, and Prof. Brickell, with consummate care, passed the blood into her arm, before it had time to cool, or even to repose for more than a few seconds. All the apparatus employed was immersed in warm water, or wrapped with heated cloths, so as to prevent any reduction of the temperature of the transfused blood. The operation was commenced at a few minutes before 1 o'clock, and was finished safely and satisfactorily, in all respects, a few minutes past that hour."

Dr. B. concludes the account as follows:

"I will merely add to this statement that the pulse, which, at half-past 12, under the influence of mental excitement, became once more appreciable, numbered 136, and immediately before the operation 125; at its termination it was 120; and three hours later it remained the same, but had acquired more fulness and strength. The voice recovered its natural tone, the face acquired color, the extremities grew warm, all nausea and hiccough ceased, and ordinary drinks were perfectly retained. From the time mentioned to the present hour, her recovery has been uninterrupted. Her health has long since ceased to be a subject of any anxiety.

"It was not doubted, at the time of the operation, that the quantity of blood injected was equal to three and a half ounces. It was afterwards ascertained, by accurate measurement of the syringe, that its capacity was not quite equal to two and a half ounces. Small as the quantity was, it yet sufficed to turn the scale in her favor.

* * * "Were the true nature and the statistical results of the operation of transfusion generally known, its performance would be demanded in many cases which are now consigned to a remediless doom. There is no fear that it will ever come to be employed as one of the common remedies. It is applicable to no pathological condition save that which is commonly called 'collapse,' induced by hemorrhage, by certain exhausting discharges, or by utter inabiltiy to receive or retain nutriment; and the only transfusion now sanctioned, either by physiology or by common sense, is that of *human venous blood into human veins, identical, as nearly as possible, with that which has been lost, and with quantity just sufficient to arrest the tendency toward death.* Prior to the year 1853, the total number of recorded cases, practiced under these conditions and restrictions, amounted to twenty-one. Of these but two died—or less than one case in ten!

"If I have any merit in this affair, it is that I have been, for many years, the earnest advocate of transfusion, in those cases where alone it is proper; that I have labored to make it binding on the consciences of medical men to practice transfusion whenever it is justifiable; that I have approved my faith in it by submitting to it, as the first instance which I am apprised of in America, a person whose life is as dear to me as my own; and that before it was attempted I seriously declared to those professional friends, heretofore named, my willingness to take upon myself all the odium that might attach to a failure. I feel that I can never sufficiently thank those gentlemen for what they did for me in this the bitterest trial of my life; and that they have thus laid upon me new obligations of fidelity to the principles of our noble profession."

We have reported this case at considerable length, because we regard it as of great importance in bringing to bear an essentially new and very powerful agent in the treatment of disease, and we cannot close without congratulating our friend, in view particularly of his relationship to the patient, on the glorious result attained.—*Med. and Surgical Reporter.*

---

TRANSLATIONS FROM RECENT EUROPEAN JOURNALS.—BY T. A. DEMME, M.D.

EFFECTS OF THE PREPARATIONS OF LEAD UPON THE INFERIOR ANIMALS.

A M. Pecart-Taschereau, a litharge manufacturer, (*Deutsche Klinik*), has investigated with some care the effects of the preparations of lead upon the inferior animals. Strange as it may seem, he soon noticed that the *Dog* is never affected with symptoms of lead poisoning. He has seen this animal roll in litharge and white lead, and afterward lick itself clean, but no evil consequences ever attracted his attention.

*The Cat*, on the contrary, is soon killed. This animal dies in convulsions, not only in establishments where litharge and white lead are prepared, but even in those places where minute quantities of lead chance to be present, in the compositor's rooms, and where newly-printed books have been stored. New ink, it is well known, always contains a certain quantity of this metal.

M. P-T. tried a very decisive experiment. He hung several cages, in each of which a cat was confined, in his factory, and in a short time, one after another, all died.

*Mice*—and the lead manufactories are infested by them—play in the litharge and white lead without any apparent evil consequences.

M. Rouart, white lead manufacturer in Clichy, remarks that *Rats*, which multiply most rapidly in his establishment, are very apt to suffer from paralysis of the lower extremities.

*The Horse*, exposed to the poisonous atmosphere of lead factories, appears to be liable to a paralysis of the muscles of the larynx. The recurrent laryngeal nerve (which conveys the motory impulses to the muscles of the larynx) appears unable to perform its proper functions, whilst the remainder of the pneumogastric nerve is totally unaffected.

The veterinary surgeon, Delanay, has performed, in these cases, tracheotomy, with the result of preserving the valuable animal, notwithstanding the local paralysis.

*Birds* soon succumb to the poison.

It has been observed that *sheep* and *goats* that feed near lead factories frequently suffer from hæmularia, and are also liable to miscarry.

It is also asserted that in such neighborhoods women are peculiarly apt to have premature labor.

### LEAD IN PHTHISIS.

According to Brockmann, no workman in lead suffers from phthisis pulmonalis, even if there is hereditary predisposition and phthisical conformation of the chest.

Is not this an indication for the use of the preparations of lead in consumption of the lungs?

Saccharum saturna (plumbi acetas) has been used in pneumonia, and pituitous catarrh, with good effects, and in the last stages of phthisis pulmonalis, it has not seldom been given as the ultimum solanum.

The properties of the lead salts are precisely those that are indicated in these cases—antiphlogistic, astringent and sedative. In consequence of these properties, lead has been called the narcoticum metallicum.—*Med. and Surgical Reporter.*

